# DAHD-YOLO: A New High Robustness and Real-Time Method for Smoking Detection †

**DOI:** 10.3390/s25051433

**Published:** 2025-02-26

**Authors:** Jianfei Zhang, Chengwei Jiang

**Affiliations:** School of Computer and Control Engineering, Qiqihar University, Qiqihar 161006, China; 2023935765@qqhru.edu.cn

**Keywords:** smoking behavior detection, robustness, reparameterization, feature pyramid network, context anchor attention

## Abstract

Recent advancements in AI technologies have driven the extensive adoption of deep learning architectures for recognizing human behavioral patterns. However, the existing smoking behavior detection models based on object detection still have problems, including poor accuracy and insufficient real-time performance. Especially in complex environments, the existing models often struggle with erroneous detections and missed detections. In this paper, we introduce DAHD-YOLO, a model built upon the foundation of YOLOv8. We first designed the DBCA module to replace the bottleneck component in the backbone. The architecture integrates a diverse branch block and a contextual anchor mechanism, effectively improving the backbone network’s ability to extract features. Subsequently, at the end of the backbone, we introduce adaptive fine-grained channel attention (AFGCA) to effectively facilitate the fusion of both overarching patterns and localized details. We introduce the ECA-FPN, an improved version of the feature pyramid network, designed to refine the extraction of hierarchical information and enhance cross-scale feature interactions. The decoupled detection head is also updated via the reparameterization approach. The wise–powerful intersection over union (Wise-PIoU) is adopted as the new bounding box regression loss function, resulting in quicker convergence speed and improved detection outcomes. Our system achieves superior results compared to existing models using a self-constructed smoking detection dataset, reducing computational complexity by 23.20% while trimming the model parameters by 33.95%. Moreover, the mAP50 of our model has increased by 5.1% compared to the benchmark model, reaching 86.0%. Finally, we deploy the improved model on the RK3588. After optimizations such as quantization and multi-threading, the system achieves a detection rate of 50.2 fps, addressing practical application demands and facilitating the precise and instantaneous identification of smoking activities.

## 1. Introduction

This article is an expanded version of a paper, which was presented at the 10th International Conference on Informatics & Applications (ICIA2024), Maca (Online), 13–15 December 2024 [1].

Smoking, a widespread harmful behavior that threatens the health of both smokers and non-smokers, can lead to a range of diseases, including esophageal cancer, lung cancer, and cardiovascular diseases [2]. Smoking not only poses a significant threat to individuals’ health but can also lead to unforeseen events like fires and industrial accidents due to smokers’ improper disposal of cigarette remnants. This presents a significant risk to public safety and can cause substantial economic losses. Therefore, detecting smoking behavior has gained importance; the goal is to detect and stop smoking behavior in time to maintain public health and safety.

Traditional detection methods have their pros and cons. Smoke sensors are cost-effective and reliable indoors but have a high alarm threshold, limiting their outdoor and well-ventilated space detection [3]. Wearable sensors are accurate in labs but lack comfort and concealment in real life [4]. As advancements in surveillance systems and vision-based analytics continue, AI-driven approaches are gaining traction. However, RGB video-based methods are affected by light and texture, and 3D convolution and two-stream networks have drawbacks like high computational complexity and complex optical flow extraction. Skeletal data are more robust. For example, in the detection of smoking behavior, high-precision facial or hand skeletal information is required, which is often accompanied by high-resolution video surveillance equipment and high computing power equipment for high-precision detection. The behavior detection-based method mainly focuses on certain smoking actions rather than the cigarette itself. But in places where smoking is strictly prohibited, such as gas stations and chemical plants, real-time performance is particularly crucial. In contrast, target detection-based methods, especially one-stage algorithms, are lightweight and offer strong real-time performance, thus holding significant application value in smoking detection.

Despite recent advancements in target detection technology, particularly in smoking behavior detection, several challenges remain. Firstly, the appearance of cigarettes changes significantly under different conditions, causing interference in accurate identification, especially in scenarios with bright or dim lighting. Secondly, data annotation processes contain inherent problems due to cigarettes being part of a whole entity rather than existing as independent objects. Generally, they are held in the mouth or between fingers, causing irrelevant objects such as fingers and lips to be introduced during the annotation process. This situation causes the model to easily deviate from the core target during the learning process, wrongly regarding irrelevant parts such as the fingers holding the cigarette as the “cigarette” target that needs to be learned and recognized, thus affecting the model’s precise comprehension of the cigarette itself. Moreover, existing models are prone to misjudgment in practical applications. Such problems occur due to the slender shapes, uniform colors, and small texture changes of objects like fingers, pens, and railings, which the model often fails to accurately distinguish, misidentifying them as cigarettes. These issues lead to challenges like incorrect positives, missed detections, and reduced identification rates, which seriously threaten the precision and dependability of detection results. Hence, adopting specific strategies is crucial to addressing these challenges, improving detection precision, and ensuring the practical implementation of smoking behavior recognition technology.

In this paper, we propose DAHD-YOLO, a model built upon the foundation of YOLOv8.

1. Firstly, we design a new module named DBCA, which serves as a substitute for the conventional bottlenecks within the C2f block of the original backbone. Through the application of the reparameterization method and context anchor’s attention [5], we optimize the model by decreasing the parameter count and computational demands, thereby strengthening the backbone’s feature extraction power.
2. Secondly, the AFGCA [6] mechanism is incorporated into the rear part of the main framework. The adaptive attention mechanism dynamically modulates the importance of features across different scales, assisting the spatial pyramid pooling fast (SPPF) module and making fused features more discriminative, thereby improving the precision of object identification across different scales. In challenging environments, it helps the model focus on key target parts while ignoring interfering background information.
3. Next, we replace the neck in the baseline model with a more efficient feature pyramid network (FPN) [7]. The FPN has fewer parameters than the path aggregation network (PAN) [8], solving information loss and resolution mismatch problems during target detection at different scales. We select efficient channel attention (ECA) [9] to efficiently achieve feature selection with low computational cost. Additionally, it effectively combines multi-scale representations while preserving contextual meanings and spatial cues at different levels.
4. Finally, we apply reparameterization to some convolutional layers in the detection head and replace the complete intersection over union (CIoU) [10] with Wise-PIoU. This enhances model network convergence during training while augmenting detection accuracy.

## 2. Related Works

Smoking behavior detection algorithms based on video analysis have become efficient technical means. By conducting real-time analysis on the video streams captured by cameras, they can accurately identify the smoking behaviors of individuals. The recognition methods based on smoking behaviors fall into two distinct categories: action recognition based on human skeletal joints and action recognition based on RGB video streams. There are three target objects for smoking detection methods based on RGB, namely, smoke, cigarettes, and the smoking behavior itself. Zhang et al. [11] aimed to detect the smoking violations of construction workers in non-smoking areas. They used the AlphaPose [12] algorithm to obtain human skeletal information and utilized the ST-GCN [13] network to extract the features of smoking actions for the preliminary identification of smoking behaviors. Compared with artificial features such as distance angles and distance ratios, it has higher robustness. However, the data needs to pass through two network models in series, resulting in relatively poor inference performance in real-time applications. Detection is carried out through multiple features of smoke. Digital image processing methods are employed to analyze and extract smoke features from cigarette images. Usually, attributes like hue and contour are utilized for smoke identification, and then the detected results are put into classifiers like support vector machine (SVM) for classification, thus realizing the detection of smoking. Lin et al. [14] achieved the detection of smoke by detecting the gray value and saturation in the oral region and through manually set thresholds. Despite having limited training data, satisfactory identification performance could be obtained. However, the shapes of smoke are often diverse. Especially in most scenarios, smoke is not easy to notice. Moreover, the features traditionally extracted and summarized are usually targeted at specific environmental scenes, and their robustness is not high. Poonam et al. [15] first used the faster region convolutional neural network (RCNN) for smoking detection, which can be applied to display warning messages in smoking scenes. It achieved relatively high precision on the self-built dataset, but the two-stage detection algorithm cannot realize real-time detection in specific scenarios. Zhang et al. [16] set four different sizes of convolution kernels according to the shape characteristics of cigarettes to extract cigarette features at different angles and scales, effectively avoiding the limitations of a single-size convolution kernel in extracting cigarette shape features. Zhao et al. [17] introduced an FPN-D-based method for detecting driver smoking behavior. This method combines FPN with the dilated convolution technique. Using images captured from the vehicle’s onboard system, it utilizes deep neural networks to generate the feature pyramid and improve the convolution operation, effectively detecting cigarettes in the driver images and thus identifying smoking behavior. Xiao et al. [18] adopted a multi-step object detection method from human faces to cigarette butts. Firstly, they used the histogram of oriented gradient (HOG) feature method to extract human faces to reduce the area to be detected for cigarette butt targets. Then, they utilized the improved YOLO algorithm with the added spatial pyramid pooling module to locate cigarette butt targets. Compared with single-step object detection, this kind of method has a lower false detection rate. However, due to the relatively long inference time, real-time detection cannot be achieved, and its application is limited in certain scenarios. Unlike the above-mentioned studies, this paper proposes DAHD-YOLO, which focuses on addressing the issues of insufficient accuracy, low recall, and real-time performance in existing smoking behavior detection models, from structural optimization and feature fusion to loss function improvement. Moreover, we deployed the improved model onto edge-side devices equipped with the RK3588 chip, successfully achieving offline and real-time smoking behavior detection.

## 3. Network Structure

### 3.1. YOLOv8 Network Structure

Among single-stage object detection architectures, YOLO remains a widely adopted approach. The latest version has reached YOLOv11 [19]. YOLOv8, on the other hand, is prevalently applied on account of its functions of instantaneous pose evaluation and object partitioning. Compared with YOLOv5, YOLOv8 usually can achieve higher accuracy in the same tasks. However, the number of parameters and the volume of computation for its corresponding n, s, m, and other models have also risen remarkably, which also results in the slower inference speed of YOLOv8 compared to the previous YOLOv5. The network component has drawn on the design concept of ELAN, specifically using C2f, which features a more abundant gradient flow. Furthermore, the detection head of YOLOv8 has been modified from the original single-branch output, which provided category and location information, to a decoupled head with two independent branches that predict the target’s classification and positional details, respectively. This improvement allows the model to conduct more efficient gradient updates for the two different tasks (classification and location) during the training process. Since the two branches operate independently and are each responsible for different prediction tasks, the problem of mutual interference between tasks that may occur under the single-branch structure is avoided, allowing the model to converge to a better parameter state more quickly, thereby enhancing the overall model’s training effectiveness and reducing the necessary training duration. Moreover, in the YOLOv8 algorithm, the TaskAlignedAssigner of TOOD [20] is directly adopted to pick training instances based on a combination of classification and localization confidence scores. The alignment metric is introduced into the loss functions of the classification and location tasks, making the network not only focus on the classification score but also on the accuracy of location during training. This approach enables a more efficient trade-off between the classification and location tasks within the model, thereby yielding more precise detection outcomes during inference.

### 3.2. DAHD-YOLO Network Structure

**Figure 1:**
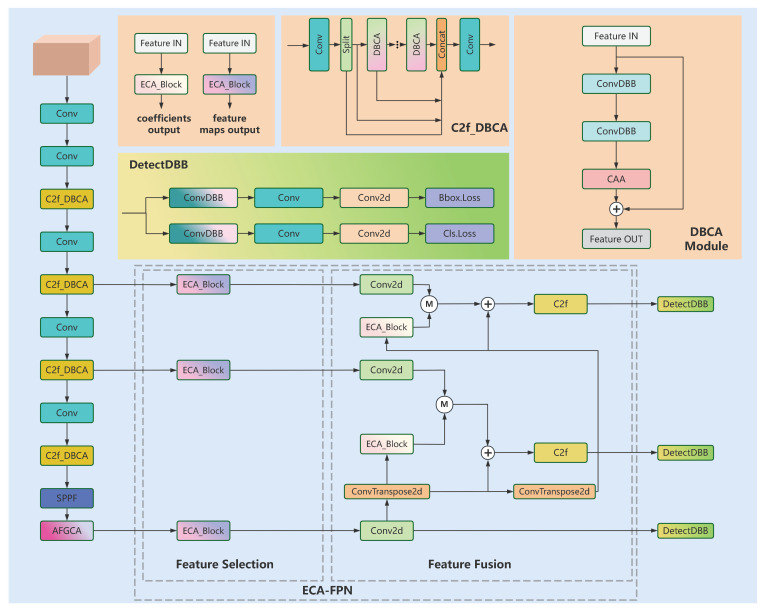
The architecture of DAHD-YOLO.

Considering the intricacy of the smoking behavior detection task, we adopt YOLOv8s as the fundamental model and present DAHD-YOLO with enhancements based on it. The network architecture diagram of DAHD-YOLO is shown in Figure 1. In the context of smoking behavior detection, the complexity of the smoking scene requires a more powerful network. The DBCA applies reparameterization and context anchor attention to optimize parameter efficiency and enhance computational performance while improving feature extraction. The ECA-FPN is composed of two main components: feature selection and feature fusion. In feature selection, ECA_Block outputs the entire feature map, while during the fusion step, ECA_Block returns the coefficients. Regarding the detection head, different from that in DBCA, we only replace the first convolutional layer of the class prediction branch and the location prediction branch. The reparameterization of the detection head and the application of Wise-PIoU significantly improve the model convergence speed and detection accuracy.

### 3.3. Feature Extraction Network

#### 3.3.1. Diverse Branch Block

Considering the complexity of cigarette detection in identifying fire-related items during the process of monitoring activities related to smoking habits. To improve the backbone network’s feature extraction capability, the diverse branch block is employed. This block captures the unique features of cigarettes more proficiently, such as their shape and texture, which are crucial for accurate smoking detection.

**Figure 2:**
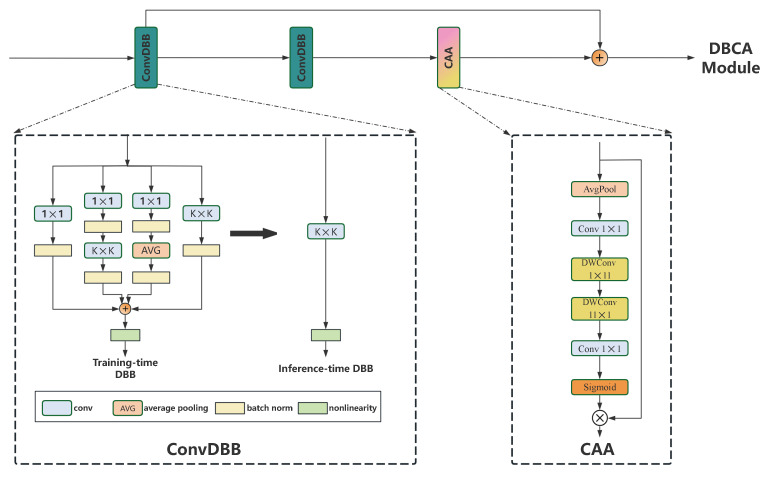
The component diagram of the DBCA module, including two sub-diagrams, namely ConvDBB and CAA.

ConvDBB adopts the idea of reparameterization from the diverse branch block (DBB) [21]. Its core idea is to use multi-branch modules (including sequential convolutions, branch addition, depth concatenation, etc.) during the training phase for training, for the purpose of obtaining optimal trait abstraction capabilities. However, during the inference stage, by calculating weights and biases, the multi-branch architecture is transformed into a corresponding single convolutional layer. In this manner, under the condition that the quantity of parameters and the speed of inference stay constant, the network can learn richer features. The enhancement of the basic Conv layer achieved by DBB is shown in the left sub-diagram, ConvDBB, of Figure 2.

We define $F\in R^{D\times C\times K\times K}$ and $b\in R^{D}$ to describe a convolutional layer. Here, *C* represents the number of input channels, *D* represents the number of output channels, and *K* represents the size of the convolutional kernel.

The function of the convolutional layer is to receive an input $I\in R^{C\times H\times W}$ and output $O\in R^{D\times H^{\prime }\times W^{\prime }}$ after processing.

⊛ represents the convolutional operation and b is replicated to $\mathrm{REP}\left(b\right)\in R^{D\times H^{\prime }\times W^{\prime }}$. The parameters $H^{\prime }$ and $W^{\prime }$ are determined by the kernel size *K*, the padding, and the stride. Formally, we have the following:

(1) O=I⊛F+REP(b)

DBB has six transformation methods, which correspond to transforming conv-BN, branch addition, sequential convolutions, depth concatenation, and average pooling into a single convolutional layer, respectively. Take the reparameterization of conv-BN into conv as an example:

For the convolutional layer containing batch normalization (BN), it usually can help accelerate the training process while reducing overfitting. We define *j* as the channel index, $\mu_{j}$ and $\sigma_{j}$ as the accumulated channel-wise mean and standard deviation, respectively. The scaling factor $\gamma_{j}$ and the bias term $\beta_{j}$ are learned parameters used for normalization. Then, feature map O, corresponding to the channel index *j*, can be expressed as follows:

(2) $$O_{j,:,:}=\left({\left(I⊛F\right)}_{j,:,:}-\mu_{j}\right)\frac{\gamma_{j}}{\sigma_{j}}+\beta_{j}$$

The homogeneity of convolutions enables the seamless fusion of BN into the preceding convolutional layer. During the training process, we still use the convolution with a BN layer. However, before the inference stage, we need to reset the values derived from the original conv-BN parameters and then store the model with a single convolutional layer for inference purposes. We define a single conv with kernel $F^{\prime }$ and bias $b^{\prime }$. Then, we can construct $F^{\prime }$ and $b^{\prime }$ for every output channel *j* from Equations (1) and (2). Formally, we have the following:

(3) $${F^{\prime }}_{j,:,:,:}\leftarrow \frac{\gamma_{j}}{\sigma_{j}}Fj,:,:,:,\;{b^{\prime }}_{j}\leftarrow -\frac{\mu_{j}\gamma_{j}}{\sigma_{j}}+\beta_{j}$$

#### 3.3.2. CAA Module

After ConvDBB, we introduce the context anchor attention [5] to build the CAA module, which improves the model’s ability to capture long-distance contextual dependencies and reinforces feature extraction in the central region. To be more specific, local region features are attained by first applying average pooling and then a 1 × 1 convolution. Afterward, instead of using traditional large-kernel convolutions, we employ two strip convolutions.

This is especially beneficial for the feature identification and extraction of cigarettes in smoking scenes. Since cigarettes are elongated items, the CAA module can effectively capture their features while minimizing parameters, resulting in enhanced performance of smoking behavior detection. Through augmenting the depth of 1D convolutions, the connection among long-range pixels is enhanced with only a marginal increment in the computation volume. The detailed network structure diagram of the CAA module is presented in the right sub-diagram of Figure 2.

### 3.4. AFGCA Feature Extraction

After extracting rich features through the DBCA in the backbone, SPP gathers feature data across various scales via pooling operations with varying granularities. In SPPF, a single fixed-size kernel is utilized for pooling rather than several pooling levels with diverse kernel dimensions, which accelerates the process but also forfeits some feature details. In smoking behavior detection, the ability to capture multi-scale features is vital. Cigarettes may appear in different sizes and poses in smoking scenes. The AFGCA mechanism can strengthen the feature representation capacity, enabling the model to better identify cigarettes in various scenarios, whether close-up or at a distance. The AFGCA mechanism promotes the interaction between overall and partial information by capturing their associations and employing cross-correlation maneuvers to acquire a correlation matrix, thereby achieving more effective feature weight distribution. The AFGCA mechanism is capable of strengthening the feature representation capacity via meticulous feature screening and the integration of comprehensive and specific features. By adding AFGCA after SPPF, we effectively combine overall and regional feature vectors using the learnable factors of the adaptive fine-grained channel attention mechanism. Further, after the final 1 × 1 Conv fusion of the SPPF structure, we enhance the adaptive distribution of weights for feature map channels. Based on comprehensive and specific details, we selectively highlight features abundant in information and restrain unproductive ones.

**Figure 3:**
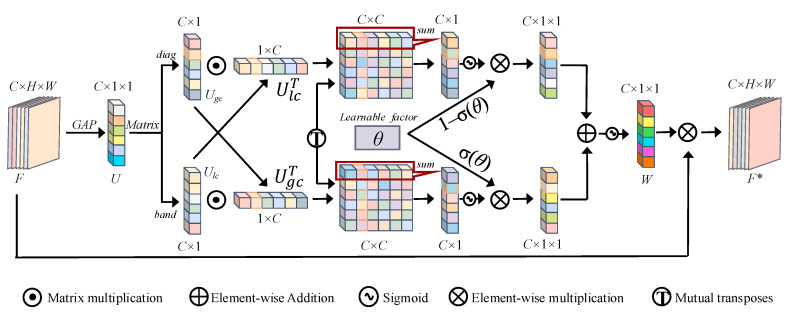
Diagram of AFGCA.

In Figure 3, $F\in R^{C\times H\times W}$ is obtained through global average pooling to obtain a feature map $U\in R^{C\times 1\times 1}$. Then, through diag matrices and band matrices, the calculations of the global feature $U_{gc}$ and the local feature $U_{lc}$ are completed with low computational costs. Next, cross-correlation operations are employed to seize the interrelation between comprehensive and specific features. Finally, the information from the rows and columns is fetched from the correlation matrix and converted into weight vectors corresponding to the comprehensive and specific information: $U_{gc}^{w}$ and $U_{lc}^{w}$. After passing through the sigmoid activation function σ(θ), they are multiplied by the learnable factors σ(θ) and 1−σ(θ), respectively, attaining dynamic fusion while evading superfluous cross-correlation maneuvers between regional and overall information. Formally, we have the following:

(4) $$U_{gc}^{w}=\sum_{j}^{c}M_{i,j},\;i\in 1,2,3\cdots c$$

(5) $$U_{lc}^{w}=\sum_{j}^{c}{\left(U_{lc}\cdot U_{gc}^{T}\right)}_{i,j}=\sum_{j}^{c}M_{i,j}^{T},\;i\in 1,2,3\cdots c$$

(6) $$W=\sigma (\sigma \left(\theta \right)\times \sigma \left(U_{gc}^{w}\right)+\left(1-\sigma \left(\theta \right)\right)\times \sigma \left(U_{lc}^{w}\right))$$

### 3.5. Feature Select and Feature Fusion

The backbone generates multi-scale feature maps rich in high-level semantic information. However, limited by the size of the feature maps, their target localization ability is relatively poor. On the contrary, low-level features provide accurate target locations yet possess restricted semantic data. A typical approach entails directly aggregating the upsampled high-level characteristics and low-level characteristics in order to endow each tier with semantic details. Nevertheless, this method lacks a feature screening process, and it is unreasonable to just aggregate the pixel numerical values of several feature strata in a coarse way.

In smoking behavior detection, due to differences in shooting angles and the varying distances between objects and the lens, there are significant scale differences among individual cigarette images. To address these challenges, we introduce the high-level screening feature pyramid network (HS-FPN) [22]. This helps the model better handle the diverse scales of cigarettes in different smoking scenes, improving the accuracy of smoking detection. In the feature selection module, through channel attention, the most representative information is extracted from each channel while reducing information loss to the greatest extent. Subsequently, filtered feature maps are obtained for fusion by applying weight information to the feature maps from corresponding scales. In the feature fusion module, high-level semantic features are mainly employed to screen low-level features, and the filtered feature maps are incorporated into the high-level feature mappings filled with rich semantic content. Then, in a top-down manner, feature layers possessing diverse scales, varying extents of semantic details, and distinct location particulars are combined in pairs, thereby enhancing the model’s feature representation capacity.

**Figure 4:**
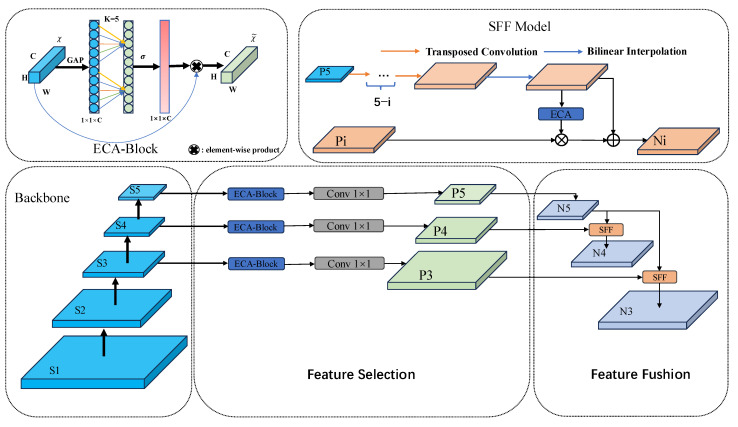
Diagram of ECA-FPN.

We use ECA-FPN to replace the original neck composed of the PAN and the FPN. It can diminish the number of parameters and the volume of computation while attaining efficient feature fusion. The model diagram of ECA-FPN is shown in Figure 4. We utilize ECA in place of channel attention to enhance the effectiveness of channel feature selection and the process of feature fusion. Channel attention fuses the features obtained by means of global average pooling and global maximum pooling; this process, which involves two pooling actions, heightens computational complexity. However, ECA directly performs global average pooling on each channel without reducing dimensionality and employs a 1D conv to attain local cross-channel interactions, reducing the volume of computation and the number of parameters, thereby enhancing computational efficiency. ECA extracts local cross-channel interactions by evaluating each channel along with its k-nearest neighbors, where the size of kernel k is directly related to the channel dimension C and automatically adapts to the range of interactions based on the channel dimension level, thereby avoiding manual parameter tuning.

### 3.6. Detect Head and Loss Function

#### 3.6.1. DetectDBB

The detection head of YOLOv8 takes the form of a separated head, applying two branches to predict the coordinates and classes of the target boxes, respectively. This enables each branch to more effectively acquire the corresponding classification or bounding box regression, reducing mutual interference between the two tasks; however, it increases the parameter and computation counts, leading to decreased inference speed. We still use the re-parameterization idea of DBB to transform the detection head. However, unlike the DBCA module in the backbone, we do not use the DBB conversion of the full branch here. The reason is that the decoupled head of YOLOv8 itself is powerful enough, and using the DBB of the full branch may introduce some unnecessary feature branches. These branches may learn some noise or features that are not helpful for the final detection task, resulting in a decrease in the final positioning and classification performance. Specifically, we use a sequential convolution block, which includes a series-connected convolution block of 1 × 1 and 3 × 3. Moreover, concise sequential convolutions can more accurately center on the crucial characteristics related to object detection, such as the boundaries, shapes, and positions of objects, reducing the interference of other irrelevant features. During the inference phase, this can be simplified to a single convolution layer, which does not affect the final inference speed while enhancing detection precision.

#### 3.6.2. Powerful IoU

To address the issue where methods based on $l_{n}$-norm loss functions overlook the correlations among the xywh variables of bounding box regression (BBR), the intersection over union (IoU) [23] is proposed, where *I* represents the overlapping region between the predicted box and the ground truth box, and *U* denotes the total area covered by both boxes. $L_{IoU}$ performs a regression on the four boundaries of the bounding box in an overall manner, overcoming the shortcomings of $l_{n}$-norm. However, in the case where the anchor box and the ground truth box have no overlap, the gradient of $L_{IoU}$ will vanish.

Although many IoU-based losses have been proposed for resolving this problem, these methods include generalized IoU (GIoU) [24], Distance-IoU (DIoU) [25], CIoU [10], SIoU [26], focal and efficient IOU (EIoU) [27], PIoU [28], and Shape-IoU [29]. However, current IoU methods have unreasonable penalty factors, and this regression method is intricate and slow, necessitating a greater number of training epochs to reach convergence.

For example, the regularization term in the CIoU loss function primarily reflects variations in the distances between center points and the aspect, but it does not account for shape discrepancies between the anchor box and the ground truth box directly, nor does it indicate variations in the dimensions of the predicted bounding box. The Powerful-IoU has a size-adaptive penalty factor. This coefficient is integrated with a mechanism that modifies the gradient in line with the status of the bounding box. This integration enables the bounding box to perform efficient and direct regression. Specifically, PIoU effectively reduces the gaps between the four edges of the bounding box and the corresponding sides of the target box, enabling a faster model convergence speed during training. Formally, we have the following:

(7) $$L_{IoU}=1-\frac{I}{U},0\leq L_{IoU}\leq 1$$

(8) $$P=\left(\frac{dw_{1}}{w_{gt}}+\frac{dw_{2}}{w_{gt}}+\frac{dh_{1}}{h_{gt}}+\frac{dh_{2}}{h_{gt}}\right)/4$$

(9) $$L_{PIoU}=L_{IoU}+1-e^{-P^{2}},0\leq L_{PIoU}\leq 2$$

In the above formula, $dw_{1}$, $dw_{2}$, $dh_{1}$, and $dh_{2}$ stand for the magnitudes of the differences between the corresponding sides of the predicted bounding box and the target bounding box. As for $w_{gt}$ and $h_{gt}$, they denote the breadth and altitude of the target bounding box, respectively. Since *P* does not depend on the dimensions of the minimum enclosing box for both the predicted and target boxes, it will not cause the predicted box to expand. When the penalty factor *P* is large, it shows that there are marked differences between the anchor box and the actual object, and $L_{IoU}$ becomes larger, thereby suppressing harmful gradients from low-quality anchor boxes. The new design of $L_{IoU}$ allows medium-quality anchor boxes to possess the largest gradients, allowing these anchor boxes to quickly regress to the area around the target box.

#### 3.6.3. Wise IoU

A large portion of current work has designed IoU loss functions based on the premise of high-quality training samples. However, real-world data are not always of high quality. In particular, when using horizontal bounding boxes to label small objects with large angular variations, such as cigarettes, it is more or less inevitable to introduce irrelevant backgrounds or other objects in some data samples when achieving absolutely perfect annotations. Enhancing the bounding box regression on such low-quality training samples would reduce the localization performance of the model. Although Focal-EIoUv1 [27] has been proposed, it is unable to make the most of the non-monotonic focusing mechanism’s capabilities. Wise-IoU with a dynamic non-monotonic focusing mechanism offers a rational allocation approach for gradient gain. This approach diminishes the dominance of high-quality anchor boxes and mitigates the adverse gradients generated by poor-quality data. As a result, Wise-IoU focuses on anchor boxes with moderate quality, which enhances the detector’s overall performance. Formally, we have the following:

(10) $$R_{WIoU}=\exp\left(\frac{{\left(x-x_{gt}\right)}^{2}+{\left(y-y_{gt}\right)}^{2}}{{\left(W_{g}^{2}+H_{g}^{2}\right)}^{*}}\right)$$

(11) $$L_{WIoUv1}=R_{WIoU}L_{IoU}$$

(12) $$L_{WIoUv2}={\left(\frac{L_{IoU}^{*}}{\bar{L_{IoU}}}\right)}^{\gamma }L_{WIoUv1}$$

$R_{WIoU}\in \left[1,e\right)$ indicates that the $L_{IoU}$ corresponding to the anchor box of ordinary quality is significantly magnified. $L_{IoU}$, which ranges between 0 and 1, substantially decreases the $R_{WIoU}$ of high-quality anchor boxes and diminishes the emphasis placed on the distance between their center points when the anchor box matches up with the target box. $W_{g}$ and $H_{g}$ represent the width and height of the smallest enclosing box. ^*^ indicates that $W_{g}$ and $H_{g}$ are detached from the computational graph to avoid the generation of gradients that impede convergence. γ is a hyperparameter with a default value of 0.5. Its role is to adjust the dynamic range of the gradient gain when using a loss function with a focal mechanism. The ratio of $L_{IoU}^{*}$ to $\bar{L_{IoU}}$ represents the outlier degree of the anchor box, where $\bar{L_{IoU}}$ represents an exponentially weighted moving average with a momentum of *m*. The normalizing factor $\bar{L_{IoU}}$ is updated dynamically to keep the value of $\frac{L_{IoU}^{*}}{\bar{L_{IoU}}}$ relatively high overall, ensuring that the rate of convergence in the final phases of training remains high.

Aimed at challenges such as complex backgrounds, large-scale variations, high-aspect ratios, etc., in the smoking detection task, we draw on the dynamic non-monotonic FM concept in Wise-IoUv2 and combine it with PIoU. We propose Wise-PIoU to suppress the harmful gradients from low-quality anchor boxes, more effectively reducing the negative impact of such anchor boxes on model training, thus achieving better positioning results and accelerating the convergence rate of the model. The loss function formula of Wise-PIoU is as follows:

(13) $$L_{WisePIoU}={\left(\frac{L_{IoU}^{*}}{\bar{L_{IoU}}}\right)}^{\gamma }L_{PIoU}$$

The PIoU has a fast-convergence characteristic and can effectively guide the bounding box regression. When combined with Wise-IoUv2, it further accelerates the convergence of the model. This combination is highly suitable for solving object-detection problems in complex tasks such as smoking detection.

**Figure 5:**
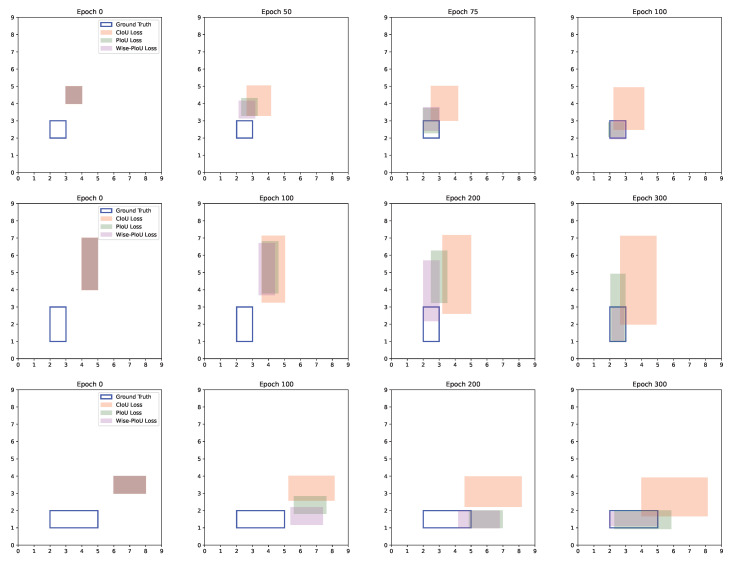
Regression results guided by different BBR losses.

In Figure 5, we carried out simulation experiments on ground truths of different shapes, including long-strip-shaped ones similar to cigarette label boxes. For ground truths of various shapes, it can be observed that Wise-PIoU can rapidly approach and fit the boundaries of the ground truth boxes. When the anchor box of Wise-PIoU has no intersection with the ground truth, Wise-PIoU and PIoU exhibit almost the same speed. However, once there is an intersection with the ground truth, the gain coefficient in front of $L_{PIoU}$ in Wise-PIoU begins to accelerate the overall convergence, thereby demonstrating a faster convergence speed than PIoU.

## 4. Experiment and Result Analysis

### 4.1. Datasets and Experimental Environment

In smoking detection, because of the deficiency of public datasets, we used a self-built one in our experiments. We collected relevant datasets and network pictures from the internet and performed label annotation and inspection by hand. The final dataset had 2810 images, split into train, val, and test sets, in an 8:1:1 ratio. The samples covered diverse lighting, sizes, and complex backgrounds, reflecting the model’s detection and generalization. The experiments were conducted on an Ubuntu 22.04 system equipped with a 3070 GPU. The versions of Python, CUDA, RKNN-Toolkit2, and PyTorch were 3.9.19, 2.1, 2.3.0, and 2.2.2, respectively. We used YOLOv8s as the benchmark network model. The hyperparameters were set as follows: 200 epochs, a batch size of 16, AMP enabled, a close_mosaic of 10, a momentum of 0.937, an initial learning rate of $1\times 10^{-2}$, and the optimizer was SGD. During the training, we employed the online data augmentation function of YOLOv8 with default parameters. It included HSV color space augmentation (involving randomly adjusting the hue, saturation, and value, respectively), geometric transformation augmentation (randomly translating the image horizontally and vertically, and randomly scaling the image), flipping the image horizontally, and so on. The hardware development board used for model deployment was Rockchip’s RK3588, equipped with an NPU, with a computing power of 6 TOPS. The NPU Driver version was 0.9.6, and the librknnrt version was 2.3.0.

### 4.2. Evaluation Indicators

Precision (P) represents the ratio of correct positive predictions among the identified objects. Recall (R) represents the ratio of correctly identified objects relative to the total count of detected items, and it can measure the probability of missed detections by the model. The computational complexity is gauged by GFLOPs, while the real-time performance is appraised by FPS. Average precision (AP) is defined as the integral of the precision–recall curve, reflecting the mean of the maximum precision values achieved across varying recall thresholds for distinct categories. Mean average precision (mAP) is computed as the arithmetic mean of the AP scores over all categories, providing a comprehensive evaluation metric for multi-class detection tasks. To elaborate further, AP serves as a critical measure in object recognition and classification tasks, quantifying the balance between precision and recall for each individual class. In this paper, since there is only one label category, which is cigarettes, in the database, the values of AP and mAP are equal.

### 4.3. Experimental Results

#### 4.3.1. C2f Block Comparative Experiment

In this section, we use different convolution modules to replace the bottleneck in C2f to highlight the feature extraction strengths of our proposed DBCA. In the experiment, we employ deformable convolution network (DCN) [30], deconvolution (DEConv) [31], DynamicConv [32], dynamic snake convolution (DySnakeConv) [33], and frequency-adaptive dilated convolution (FADC) [34].

As shown in Table 1, after incorporating our proposed DBCA module, the proposed model attained the top mAP50 and mAP50:95 values—82.9% and 46.4%, respectively. Compared to the initial C2f, there is a decrease of 0.7 M in the number of parameters and 2.1 G in GFLOPs; mAP50 and mAP50:95 are improved by 2% and 0.9%, respectively.

#### 4.3.2. Feature Fusion Comparative Experiment

**Figure 6:**
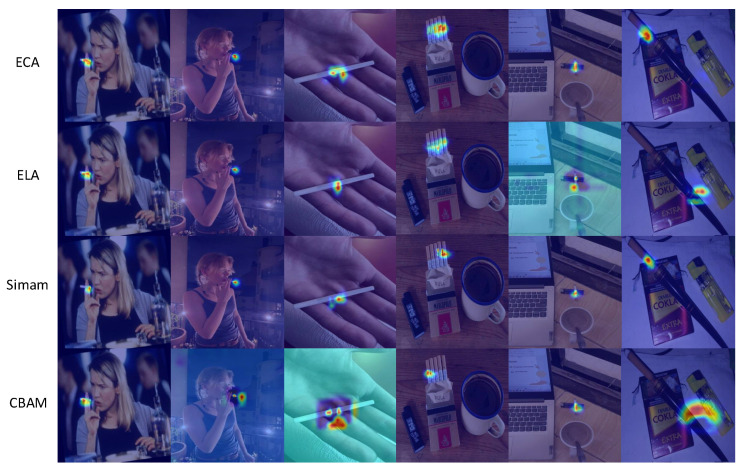
Comparison of feature visualization after adding different attention mechanisms in FPN.

We chose several typical attention mechanisms to contrast the feature selection module’s efficacy within the FPN network, including efficient squeeze-and-excitation (EfficientSE) [23], efficient local attention (ELA) [35], convolutional block attention module (CBAM) [36], squeeze-and-excitation attention (SEAttention) [37], and a simple, parameter-free attention module (SimAM) [38]. We employ EigenGradCAM [39] to produce and compare heatmaps with the incorporation of different attentions. For heatmaps, the warm and cool colors represent the level of attention the model pays. The warmer the color of an area, the more attention the model gives to that area. The detection performances of different attention mechanisms are shown in Table 2 and the feature heatmaps of some attention mechanisms are presented in Figure 6.

From Table 2, it can be seen that ECA has the highest mAP50 and mAP50:95, as well as the highest recall rate (79.8%). Moreover, both its parameter quantity and computational load are at their lowest levels. This is of crucial importance for one-stage real-time detection models. It can be seen from the figure that ECA delivers good overall performance and relatively good detection effects under different light conditions, scales, and dense targets. From Figure 6, it is evident that when compared to other attention mechanisms, the incorporation of ECA enables the FPN to perform better in feature selection and fusion. With the introduction of ECA, the model focuses more on the cigarette objects themselves rather than fingers or other objects with similar shapes.

#### 4.3.3. Comparison of Loss Functions

To confirm the efficacy of the proposed Wise-PIoU, a comparison and analysis were carried out on existing loss functions including GIoU [24], DIoU [25], CIoU [10], SIoU [26], EIoU [27], PIoU [28], and Shape-IoU [29].

The evaluation indicators include mAP50, mAP50:95, and the convergence speed of the model. By means of a thorough examination of the experimental outcomes, our intention is to demonstrate the superiority of the Wise-PIoU loss function in augmenting the precision and convergence rates of object detection tasks.

**Figure 7:**
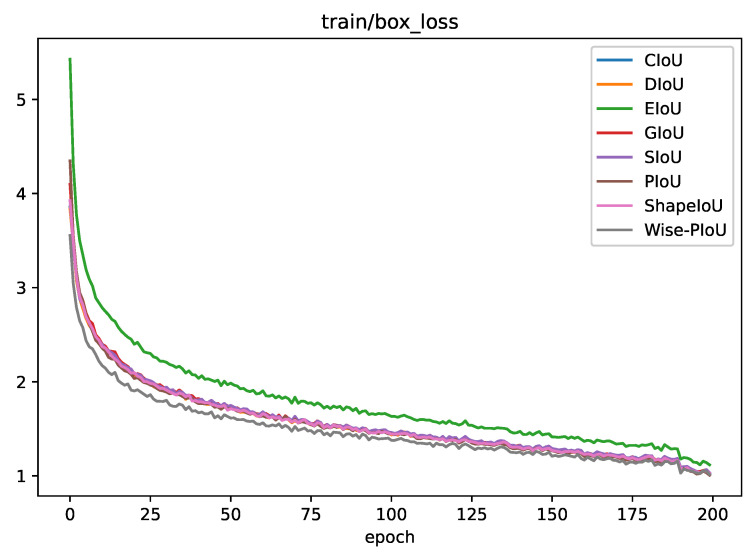
Comparison of convergence speeds between Wise-PIoU and traditional loss functions.

From Table 3, it can be seen that PIoU and CIoU achieved the highest values of mAP50:95 and mAP50 among the common IoU loss functions, respectively. Wise-PIoU achieves the highest mAP50 and mAP50:95, reaching 86.0% and 47.3%, respectively. It can be seen from Figure 7 that with the application of Wise-PIoU, a faster convergence speed can be attained in terms of box_loss and dfl_loss. Upon a thorough assessment of the experimental outcomes, it can be ascertained that the proposed Wise-PIoU loss function is efficacious in boosting both the precision and convergence tempos of object detection tasks.

#### 4.3.4. Ablation Study

To evaluate the effectiveness of the proposed improved modules of DAHD-YOLO, we conducted 10 groups of ablation experiments. The experimental results are shown in Table 4.

Table 4 shows that replacing the bottleneck in C2f of the backbone with the DBCA module improves the model’s mAP50 and mAP50:95 by 2.0% and 0.9%, respectively, suggesting more effective feature extraction with a 6.6% reduction in parameters. Adding the AFGCA module at the end of the backbone slightly increases mAP50 by 0.4% and increases mAP50:95 by 0.2% compared to the baseline, with minimal impacts on other metrics. Experiment 4 shows that using FPN with ECA_Block to replace the original neck significantly improves accuracy; mAP50 and mAP50:95 increase by 3.4% and 2.5%, respectively, with a 37.6% reduction in parameters and a 15.8% decrease in GFLOPs, while maintaining a relatively stable FPS. Experiment 5 shows that using the reparameterized DetectDBB detection head changes mAP50 and mAP50:95 by 1.5% and 1.4%, respectively. When Wise-PIoU is used to replace the default CIoU, it changes the mAP50 by 0.6% compared to the baseline. From Experiment 7 to Experiment 8, the value of mAP50 decreases by 0.3%, but mAP50:95 increases by 0.8%. Meanwhile, in terms of percentage, the computational complexity declines by 39%, the parameter quantity drops by 17%, and the model size shrinks by 33%. The FPS also increases from 244 to 272. Integrating all the improvement strategies significantly increases mAP50 and mAP50:95 values by 5.1% and 1.8%, respectively, maintaining a relatively stable FPS. Computational load and parameter count are effectively managed, with a 34.9% decrease in parameter quantity and a 23.2% decrease in GFLOPs value. This experiment demonstrates that each modification optimizes the DAHD-YOLO model’s performance in different ways.

#### 4.3.5. Comparison of Detection Models

We, respectively, employ common object detection models to make comparisons with our proposed DAHD-YOLO. The comparison models include YOLOv5s, YOLOv8s, YOLOv9s, YOLOv10s, YOLOv11s, RTMDet [40], RT-DETR [41], Faster-RCNN [42], Mask-RCNN [43], and Cascade-RCNN [44]. The experimental results are presented in Table 5.

As shown in Table 5, our DAHD-YOLO model outperforms various SOTA detection models. In accuracy, its mAP50 reaches 86.0%, surpassing many mainstream models like YOLOv5s (73.6%) and RTMDet (83.1%). In terms of parameters, with 7.24 M, it is on par with efficient models such as YOLOv5s (7.01 M) and YOLOv9s (7.17 M), far less than large-scale ones. Overall, DAHD-YOLO’s balanced excellence in these aspects showcases its superiority for practical object detection.

#### 4.3.6. Verifying the Role of the AFGCA Module

To verify the capacity of AFGCA to dynamically integrate worldwide and regional information and promote the self-adaptive apportionment of channel weights, this experiment employed EigenGradCAM to produce and compare heatmaps prior to and subsequent to the incorporation of the AFGCA. For heatmaps, the warm and cool colors represent the level of attention the model pays. The warmer the color of an area, the more attention the model gives to that area.

**Figure 8:**
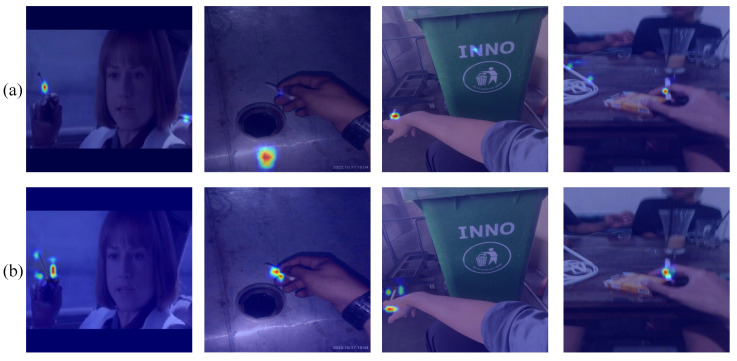
Comparison of heatmap effects: (**a**) YOLOv8 base model; (**b**) improved model with AFGCA.

From Figure 8, we can observe that after incorporating the AFGCA mechanism, the model can combine local and global information in a dynamic and fine-grained manner, facilitating the adaptive allocation of channel weights. As shown in the figure, after adding the AFGCA attention mechanism, the model places greater emphasis on the local characteristics near the face and hands, thus reducing the extraction of local information that causes interference from distant areas, such as light and shadow, printed words, and power strips. It can also be concluded from the third experiment, as shown in Table 4 of the ablation experiment, that after adding the AFGCA module following SPPF, mAP50 and mAP50:95 increased by 0.4% and 0.2%, respectively, accompanied by minor increases in the computation volume and the parameter count. As a result, the FPS slightly dropped from 289.7 to 287.9. In the seventh experiment, when the DBCA module and the AFGCA module were applied simultaneously, the mAP50 of the model witnessed a 4.3% increment in contrast to the initial model, accompanied by reductions in computational load and parameter quantity, demonstrating a significant improvement in accuracy.

#### 4.3.7. Deployment of DAHD-YOLO on RK3588

We utilize the Orange Pi 5 Plus equipped with the RK3588 SoC as our deployment platform. Compared to less powerful development boards like Raspberry Pi, RK3588 offers substantial computational capabilities with up to 6 TOPS. It supports model conversion and deployment across multiple frameworks such as PyTorch and TensorFlow.

Initially, the trained model’s corresponding pt weight file is converted to the more versatile ONNX format, facilitating easier execution in edge environments. Subsequently, the model weights undergo quantization, converting the high-precision float32 values from the training phase to lower-precision formats such as half-precision floats (FP16) or the 8-bit integer (INT8). This enhances computational efficiency and reduces memory requirements. Specifically, we employ linear asymmetric quantization, quantizing each channel of the network layers independently, with each channel having its own quantization parameters. This approach better accommodates inter-channel variations and results in improved quantization effects. We establish a calibration set, comprising 200 randomly selected images from the training data, to calculate the quantization range for activation values. The quantized model is then deployed onto the development board. After multiple tests, the INT8 optimized model’s single-thread inference time per frame is 0.1162 s, which is insufficient for real-time surveillance camera monitoring at 25 frames per second. Benefiting from its advanced architecture, the RK3588 integrates three NPU cores, each delivering a computational power of 2 TOPS, supporting modes such as three-core cooperative operation, dual-core cooperation, and independent operation. By utilizing a thread pool for acceleration, we optimize the entire detection process through multithreading. The performance of the model after thread pool optimization is presented in Table 6.

In Table 6, benefiting from the efficient scheduling of the NPU under the thread pool, the inference latency of the quantized model for a single image with a resolution of 1280 × 720 reaches 79.1 ms. At a resolution of 1920 × 1080, the non-quantized improved model can achieve a frame rate of 24.2 FPS, which is the standard rate of ordinary surveillance cameras. Moreover, at this time, the power consumption of the entire inference hardware platform is less than 10 W. In contrast, the total hardware power consumption of the inference platform with a 3070 graphics card reaches about 200 W. Compared with traditional high-energy-consumption computing hardware, the RK3588 is more energy-efficient and suitable for the deployment of downstream tasks such as smoking behavior detection.

#### 4.3.8. Visual Analysis

**Figure 9:**
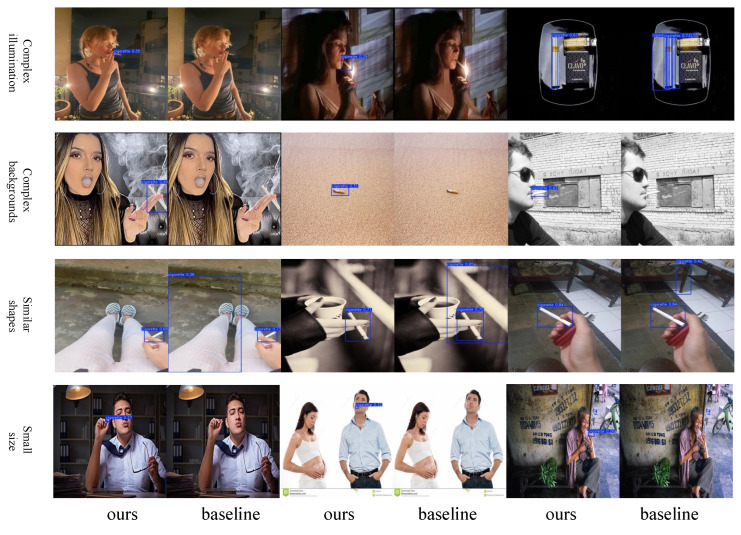
Comparison of the detection effects of the original model and the improved model in complex scenarios.

The smoking detection task under complex external conditions is affected by various factors, including different lighting conditions, complex backgrounds, similar objects, small sizes, and so on. To address these issues, we evaluated the detection performance of the improved model under the above-mentioned different scenarios, as shown in Figure 9. It can be seen that under complex lighting conditions, our model can accurately locate cigarettes rather than light spots with similar shapes. The model can well distinguish cigarettes from other objects with slender shapes such as railings and table legs. The model also has a better detection effect on relatively small-sized cigarettes compared to the original baseline model. The results demonstrate that the improved model can detect cigarette targets in different scenarios more accurately compared to the original baseline model.

#### 4.3.9. Model Generalization Ability

We selected a public dataset named “cigarettes butts yolov8 dataset” [46] on Kaggle to verify the generalization ability of the model we proposed. This dataset contains 2167 observations split into 3 different folders (train, valid, test). We retrained the YOLO-series models on this dataset and evaluated the model’s performance using the valid set. The detailed experimental results are presented in Table 7. In the table, our improved model achieved the highest recall and mAP, indicating that it can also exhibit excellent generalization performance on other smoking datasets.

## 5. Conclusions

In this article, we propose a model named DAHD-YOLO, which is used to accomplish the smoking behavior detection task in complex scenarios. First, we replace the C2f block in the backbone with a DBCA module, which enhances feature extraction while reducing parameters and computation using reparameterization and context anchor attention. Then, we incorporate the AFGCA module after the backbone to assist the SPPF in generating more distinguishable features and enhancing detection accuracy. Additionally, we replace the original neck with a more efficient feature pyramid network, reducing parameters and solving information loss and resolution mismatch issues. We utilize the efficient channel attention mechanism to achieve feature selection and feature fusion at different scales. Finally, we apply reparameterization to part of the convolutional layers in the detection head and change the loss function to Wise-PIoU, speeding up convergence and enhancing accuracy without increasing computation and parameters. We designed experiments, including contrast experiments, ablation experiments, and visualization heatmaps, to verify the high efficiency of our proposed model and its high robustness in complex environments. The experimental data illustrate that our model achieves superior detection outcomes with comparatively lower resource utilization and enhanced accuracy metrics. It can proficiently cope with the intricate scenarios within our self-constructed dataset for smoking behavior detection.

Although our model does not involve a facial detection module, smoking detection models may raise privacy concerns if misused. Data anonymization based on differential privacy and strict limitations on usage scenarios can effectively address this issue. In our experiments, we recognize the potential biases present in the dataset, which may influence the model’s generalization capabilities. While we employed a fixed random seed to ensure reproducibility and facilitate comparisons of improvements, this approach also imposes limitations on the statistical significance of our results. Consequently, the observed performance enhancements might be skewed. Additionally, the dataset may not entirely capture the diversity of real-world application scenarios, so the improvements reported may not accurately reflect the model’s performance in practical settings. To address these concerns and provide a more comprehensive evaluation of the model’s significant improvements, future work will implement cross-validation and paired *t*-tests. These statistical methods will enable us to rigorously assess the effectiveness of model enhancements across various experimental conditions, minimizing the impact of bias.

To further boost the model’s performance in real-world applications, we plan to integrate more diverse real-world data that better represent varying lighting conditions and background complexities. We will also explore adaptive fine-tuning techniques to continuously optimize the model in new environments, ensuring better alignment with practical deployment scenarios. Beyond smoking behavior detection, our proposed DAHD-YOLO can facilitate transfer learning for a variety of tasks simply by substituting the relevant training datasets. For instance, deploying the RK3588 as the hardware platform in an in-vehicle computer allows for efficient training of DAHD-YOLO to tackle various behavior detection challenges, including answering phone calls and recognizing distracted driving. The model’s increased structural complexity has resulted in a decline in inference speed. The CAA in the DBCA module relies on an average pooling operation, which, although devoid of trainable parameters, still imposes a computational load, particularly with the relatively large feature maps in the initial layers of the backbone. To mitigate this issue, we plan to replace the average pooling and subsequent 1 × 1 convolution with dilated convolutions, aiming to reduce computational demands while maintaining accuracy. Additionally, we will investigate mixed-precision quantization and distillation techniques to further enhance both the accuracy and inference speed of the model at the edge side, ensuring it remains efficient for real-time applications.

## Figures and Tables

**Table 1:** Performance comparison of different Conv modules in C2f.

Module	Params/M	GFLOPs/G	mAP50/%	mAP50:95/%
C2f_DCNv3	10.4	26.4	79.9	43.3
C2f_DEConv	11.1	28.5	81.3	44.1
C2f_DynamicConv	14.0	25.6	81.8	45.1
C2f_DySnakeConv	12.7	30.6	80.0	45.3
C2f_FADC	11.2	27.0	81.6	43.1
C2f	11.1	28.4	80.9	45.5
C2f_DBB	11.1	28.4	82.7	45.9
C2f_DBCA	10.4	26.3	82.9	46.4

**Table 2:** Model performances after applying different attention mechanisms.

Method	Params/M	GFLOPs/G	P/%	R/%	mAP50/%	mAP50:95/%
SEAttention	6.5	21.8	88.6	72.4	82.4	45.8
EfficientSE	6.9	21.8	82.4	78.1	82.1	45.6
SimAM	6.5	21.8	86.5	75.6	83.3	45.6
CBAM	6.5	21.8	83.1	71.1	79.3	43.3
ELA	9.1	22.1	86.6	73.5	81.2	44.7
ECA	6.5	21.8	86.0	79.8	84.9	47.0

**Table 3:** Comparison of different loss functions.

Method	mAP50/%	mAP50:95/%	Method	mAP50/%	mAP50:95/%
CIoU	85.5	47.2	Wise-CIoU	84.9	46.8
DIoU	84.1	45.1	Wise-DIoU	83.3	47.1
EIoU	84.3	46.7	Wise-EIoU	82.6	47.0
GIoU	82.8	46.6	Wise-GIoU	82.0	44.1
PIoU	83.8	47.4	Wise-PIoU	86.0	47.3
SIoU	82.9	45.4	Wise-SIoU	84.5	47.1
Shape-IoU	81.3	44.4	Wise-Shape-IoU	82.4	45.5

**Table 4:** Ablation study of DAHD-YOLO.

	YOLO	DB	AF	ECA-FPN	DBB	Wise-PIoU	mAP	mAP	Params	GFLOPs/G	Size/M	FPS
	V8s	CA	GCA		Detect		50/%	50:95/%				
1	✓						80.9	45.5	11,125,971	28.4	21.5	289.7
2	✓	✓					82.9	46.4	10,390,467	26.3	23.8	239.6
3	✓		✓				81.3	45.7	11,388,633	28.4	22.0	287.9
4	✓			✓			84.3	48.0	6,945,644	23.9	13.5	330.0
5	✓				✓		82.4	44.1	13,367,699	28.5	22.8	289.1
6	✓					✓	81.5	44.3	11,125,971	28.4	21.5	287.9
7	✓	✓	✓				85.2	46.2	10,653,129	26.3	24.3	243.5
8	✓	✓	✓	✓			84.9	47.0	6,472,802	21.8	16.3	271.8
9	✓	✓	✓	✓	✓		85.5	47.2	7,237,922	21.8	16.5	271.7
10	✓	✓	✓	✓	✓	✓	86.0	47.3	7,237,922	21.8	16.5	271.7

✓ indicates that the module was added to the network.

**Table 5:** Comparison of common detection models.

Method	Params/M	GFLOPs/G	Size/M	mAP50/%	mAP50:95/%	FPS
YOLOv5s	7.01	15.8	12.6	73.6	38.9	390.3
YOLOv8s	11.13	28.4	21.5	80.9	45.5	289.7
YOLOv9s	7.17	26.7	14.5	81.7	46.3	223.4
YOLOv10s	7.22	21.4	15.8	76.6	46.4	282.8
YOLOv11s	9.41	21.3	18.3	80.7	44.6	290.7
RTMDet	8.86	14.75	34.1	83.1	46.7	175.0
RT-DETR	19.87	56.9	38.6	83.4	46.9	141.5
Faster-RCNN	41.35	134	158.1	81.6	43.6	29.1
Cascade-RCNN	69.15	162	264.1	83.3	44.9	22.5
Mask-RCNN ^1^	47.80	267	183.6	81.7	38.8	20.2
DAHD-YOLO	7.24	21.8	16.5	86.0	47.3	271.7

^1^ The Mask-RCNN uses Swin Transformer [45] as its backbone.

**Table 6:** Performance results of the model deployed on RK3588.

Models	Quantization	Size/M	FPS			mAP50/%	Inference-Time/ms	Power/W
			720P	1080P	1440P			
YOLOv8s	FP16	24.1	32.9	28.1	25.2	73.6	131.9	7
YOLOv8s	INT8	12.6	70.9	66.6	57.3	73.0	56.3	9
DAHD-YOLO	FP16	15.6	26.9	24.2	22.2	78.76	116.2	6
DAHD-YOLO	INT8	8.8	50.2	43.8	37.7	74.36	79.1	7

**Table 7:** Performance comparison of the different models based on the Kaggle dataset.

Model	Params/M	Size/M	P/%	R/%	mAP50/%	mAP50:95/%
YOLOv5s	7.01	12.6	76.9	74.2	80.3	46.3
YOLOv8s	11.13	21.5	83.1	64.8	74.7	45.8
YOLOv9s	7.17	14.5	88.7	63.7	76.6	47.5
YOLOv10s	7.22	15.8	84.0	70.7	81.2	49.0
YOLOv11s	9.41	18.3	82.3	71.7	79.5	50.3
DAHD-YOLO	7.24	16.5	83.1	77.2	82.9	51.4

## Data Availability

Data are contained within the article.
